# Supplementary figures and images for: Specialized 16SrX phytoplasmas induce diverse morphological and physiological changes in their respective fruit crops
Source: PLoS Pathog. 2021 Mar 25;17(3):e1009459. doi: 10.1371/journal.ppat.1009459 (PMC8023467; doi:10.1371/journal.ppat.1009459)

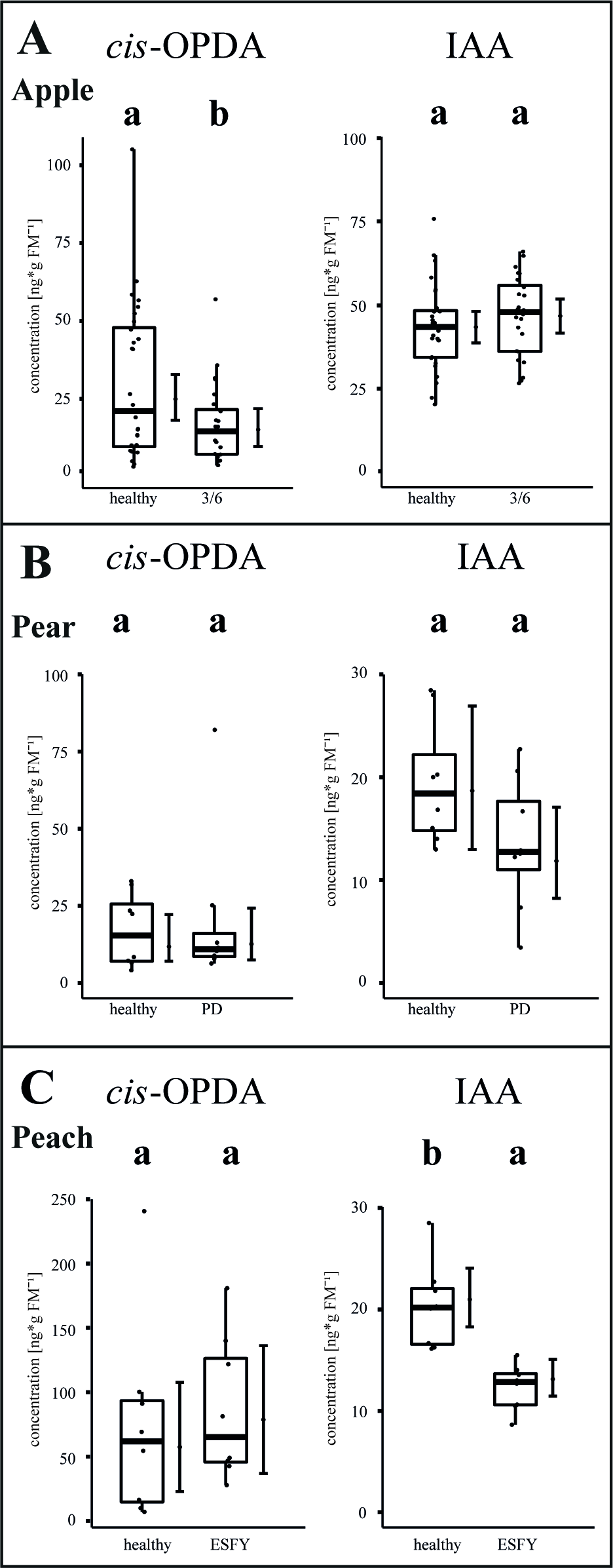

Supplement: S1 Fig — Phytohormone concentrations of cis-12-oxo-phytodienoic acid (cis-OPDA) and indole-3-acetic acid (IAA) in uninfected and phytoplasma infected (a) apple, (b) pear and (c) peach trees. Amounts of phytohormones were measured in the leaves of healthy and phytoplasma infected (a) apple, (b) pear and (c) peach. For apple, a virulent accession (3/6) was considered inducing apple proliferation (AP). Pear trees showed pear decline (PD) and peach trees showed the European stone fruit yellows (ESFY). Box-whisker plots with median as lines and jittered raw values as closed circles (corresponding to each measurement). Boxes represent the interquartile range (IQR) and whiskers extend to 1.5*IQR. Bars represent the 95% confidence intervals with the estimated marginal means obtained from linear models as dots (both back transformed to the response scale). Letters indicate statistical differences between EMMs of groups at the 0.05 significance level. (TIF) [file ppat.1009459.s001.tif]

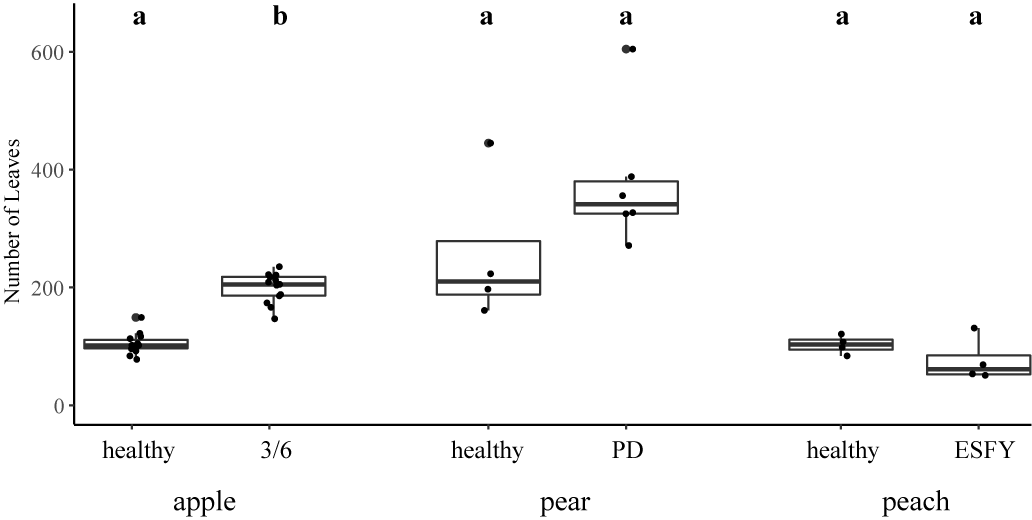

Supplement: S2 Fig — For apple, a virulent accession (3/6) was considered inducing apple proliferation (AP). Pear trees showed pear decline (PD) and peach trees showed the European stone fruit yellows (ESFY). Box-whisker plots with median as lines and jittered raw values as closed circles (corresponding to each measurement). Boxes represent the interquartile range (IQR) and whiskers extend to 1.5*IQR. Bars represent the 95% confidence intervals with the estimated marginal means obtained from linear models as dots (both back transformed to the response scale). Letters indicate statistical differences between EMMs of groups at the 0.05 significance level. (TIF) [file ppat.1009459.s002.tif]
