# Supplementary material for: Specialized 16SrX phytoplasmas induce diverse morphological and physiological changes in their respective fruit crops
Source: PLoS Pathog. 2021 Mar 25;17(3):e1009459. doi: 10.1371/journal.ppat.1009459 (PMC8023467; doi:10.1371/journal.ppat.1009459)
Supplement: S1 Table — Mean phytohormone concentrations (ng gFM-1) extracted from leaf material of Malus domestica, Pyrus communis and Prunus persica. Diverse phytohormones were detected—salicylic acid (SA), jasmonic acid-iso leucine (JA-Ile), jasmonic acid (JA), abscisic acid (ABA), 12-Oxo-phytodienoic acid (cis-OPDA) and indole acetic acid (IAA). Different letters indicate significant differences between phytoplasma infected and uninfected trees compared among the species. AP = apple proliferation, PD = pear decline, ESFY = European stone fruit yellows, npl = amount plants, nl = amount leaves. (DOCX) [file ppat.1009459.s003.docx]

**S1 Table. Details of mass spectrometer settings for analysis of phytohormones by LC-MS/MS [HPLC 1260 (Agilent Technologies)-QTRAP6500 (SCIEX)] using multiple reaction monitoring (MRM) in negative ionisation mode. RT= retention time, RF = response factor, DP = declustering potential, EP = entrance potential, CE = collision energy, CXP = collision cell exit potential.**

| **Q1 (m/z)** | **Q3 (m/z)** | **RT (min)** | **Compound** | **Internal std** | **RF** | **DP (V)** | **EP (V)** | **CE (V)** | **CXP (V)** |
| --- | --- | --- | --- | --- | --- | --- | --- | --- | --- |
| 136.93 | 93 | 3.3 | SA | D4-SA | 1.0 | -20 | -8 | -24 | -7 |
| 263 | 153.2 | 3.4 | ABA | D6-ABA | 1.0 | -20 | -12 | -22 | -2 |
| 209.07 | 59 | 3.6 | JA | D6-JA+D5-JA | 1.0 | -20 | -9 | -24 | -2 |
| 322.19 | 130.1 | 3.9 | JA-Ile | D6-JA-Ile+D5-JA-Ile | 1.0 | -50 | -4.5 | -30 | -4 |
| 290.9 | 165.1 | 4.6 | OPDA | D6-JA+D5-JA | 1.0 | -20 | -12 | -24 | -2 |
| 140.93 | 97 | 3.3 | D4-SA |  |  | -20 | -8 | -24 | -7 |
| 269 | 159.2 | 3.4 | D6-ABA |  |  | -20 | -12 | -22 | -2 |
| 215 | 59 | 3.6 | D6-JA |  |  | -20 | -9 | -24 | -2 |
| 214 | 59 | 3.6 | D5-JA |  |  | -20 | -9 | -24 | -2 |
| 328.19 | 130.1 | 3.9 | D6-JA-Ile |  |  | -50 | -4.5 | -30 | -4 |
| 327.19 | 130.1 | 3.9 | D5-JA-Ile |  |  | -50 | -4.5 | -30 | -4 |
