# Supplementary material for: Specialized 16SrX phytoplasmas induce diverse morphological and physiological changes in their respective fruit crops
Source: PLoS Pathog. 2021 Mar 25;17(3):e1009459. doi: 10.1371/journal.ppat.1009459 (PMC8023467; doi:10.1371/journal.ppat.1009459)
Supplement: S2 Table — Length and width of leafs and diameters of midribs were measured from phytoplasma infected and non-infected Malus domestica, Pyrus communis and Prunus persica. The leaf ratio was calculated by dividing the leaf length by the leaf width, the midrib ratio is defined as the ratio between the midrib diameter and the leaf width. All parameters were compared between phytoplasma infected and non-infect trees within each plant species. (DOCX) [file ppat.1009459.s004.docx]

**S2 Table. Specification and results of statistical models used for analysis of morphology parameters.** Length and width of leafs and diameters of midribs were measured from phytoplasma infected and non-infected *Malus domestica*, *Pyrus communis* and *Prunus persica.* The leaf ratio was calculated by dividing the leaf length by the leaf width, the midrib ratio is defined as the ratio between the midrib diameter and the leaf width. All parameters were compared between phytoplasma infected and non-infect trees within each plant species.

|  | **Parameter** | **Typ of analysis** | **Error**  **distribution** | **Link-Funktion** | **Chisq /**  ***F*-value** | ***P*-value** |
| --- | --- | --- | --- | --- | --- | --- |
| **Apple** | leaf ratio | Glmer | Gamma | inverse | *χ²*= 1.813 | 0.178 |
|  | leaf length | Glmer | Gamma | log | *χ²*= 13.668 | 0.0002 |
|  | leaf width | Glmer | Gamma | inverse | *χ²*= 18.123 | <.0001 |
|  | midrib ratio | Lmer | - |  | *F*=1.005 | 0.329 |
|  | midrib diameter | Lmer | - |  | *F*=21.795 | 0.0002 |
| **Pear** | leaf ratio | Glmer | Gamma | log | *χ²*= 7.796 | 0.005 |
|  | leaf length | Glmer | Gaussian | log | *χ²*= 0.031 | 0.859 |
|  | leaf width | Glmer | Gamma | identity | *χ²*= 4.784 | 0.029 |
|  | midrib ratio | Glmer | Gamma | inverse | *χ²*= 2.796 | 0.094 |
|  | midrib diameter | Lmer | - | - | *F*=1.499 | 0.267 |
| **Peach** | leaf ratio | Glmer | Gamma | log | *χ²*= 4.269 | 0.039 |
|  | leaf length | Glmer | Gamma | inverse | *χ²*= 0.863 | 0.353 |
|  | leaf width | Glmer | Gamma | identity | *χ²*= 2.136 | 0.144 |
|  | midrib ratio | Glmer | Gaussian | log | *χ²*= 8.583 | 0.003 |
|  | midrib diameter | Lmer | - | - | *F*=0.035 | 0.858 |
