# Supplementary material for: Specialized 16SrX phytoplasmas induce diverse morphological and physiological changes in their respective fruit crops
Source: PLoS Pathog. 2021 Mar 25;17(3):e1009459. doi: 10.1371/journal.ppat.1009459 (PMC8023467; doi:10.1371/journal.ppat.1009459)
Supplement: S3 Table — Areas of the vascular bundle, xylem, phloem and ten sieve elements were measured from phytoplasma infected and non-infected Malus domestica, Pyrus communis and Prunus persica. The ratio between xylem area and phloem area and of SE to phloem were calculated. All parameters were compared between phytoplasma infected and non-infect trees within each plant species. (DOCX) [file ppat.1009459.s005.docx]

**S3 Table. Specification and results of statistical models used for analysis of vascular morphology***.* Areas of the vascular bundle, xylem, phloem and ten sieve elements were measured from phytoplasma infected and non-infected *Malus domestica*, *Pyrus communis* and *Prunus persica*. The ratio between xylem area and phloem area and of SE to phloem were calculated. All parameters were compared between phytoplasma infected and non-infect trees within each plant species.

|  | **Parameter** | **Typ of analysis** | **Error**  **distribution** | **Link-Funktion** | **Chisq /**  ***F*-value** | ***P*-value** |
| --- | --- | --- | --- | --- | --- | --- |
| **Apple** | vascular bundle area | Lmer | - | - | *F*=20.922 | <.0001 |
|  | xylem area | Lmer | - | - | *F*=34.344 | <.0001 |
|  | phloem area | Lmer | - | - | *F*=13.189 | 0.0008 |
|  | SE area | Glmer | Gamma | log | *χ²*= 68.881 | <.0001 |
|  | xylem/phloem | Glmer | Gamma | identity | *χ²*= 11.916 | 0.0005 |
|  | SE/phloem | Glmer | Gamma | log | *χ²*= 0.0359 | 0.850 |
| **Pear** | vascular bundle area | Glmer | Gamma | log | *χ²*= 0.2005 | 0.654 |
|  | xylem area | Lmer | - | - | *F*=1.2709 | 0.303 |
|  | phloem area | Glmer | Gamma | log | *χ²*= 0.0417 | 0.838 |
|  | SE area | Glmer | Gamma | inverse | *χ²*= 0.2732 | 0.601 |
|  | xylem/phloem | Glmer | Gamma | identity | *χ²*= 1.2356 | 0.266 |
|  | SE/phloem | Glmer | Gamma | log | *χ²*= 0.5542 | 0.457 |
| **Peach** | vascular bundle area | Glmer | Gamma | log | *χ²*= 0.0024 | 0.961 |
|  | xylem area | Lmer | - | - | *F*=0.3666 | 0.567 |
|  | ühloem area | Glmer | Gamma | log | *χ²*= 0.1948 | 0.659 |
|  | SE area | Glmer | Gamma | log | *χ²*= 11.705 | 0.0006 |
|  | xylem/phloem | Glmer | Gamma | identity | *χ²*= 1.6635 | 0.197 |
|  | SE/phloem | Glmer | Gaussian | log | *χ²*= 4.512 | 0.034 |
