# Supplementary material for: Specialized 16SrX phytoplasmas induce diverse morphological and physiological changes in their respective fruit crops
Source: PLoS Pathog. 2021 Mar 25;17(3):e1009459. doi: 10.1371/journal.ppat.1009459 (PMC8023467; doi:10.1371/journal.ppat.1009459)
Supplement: S4 Table — The phloem mass flow velocity, the refractive index, the dynamic viscosity and density of phloem sap were measured from phytoplasma infected and non-infected Malus domestica, Pyrus communis and Prunus persica. The volumetric flow rate was calculated by multiplication of measured mass flow velocity with the median area of ten sieve elements. All parameters were compared between phytoplasma infected and non-infect trees within each plant species. (DOCX) [file ppat.1009459.s006.docx]

**S4 Table. Specification and results of statistical models used for analysis of the translocation in phloem sieve elements and physicochemical parameters***.* The phloem mass flow velocity, the refractive index, the dynamic viscosity and density of phloem sap were measured from phytoplasma infected and non-infected *Malus domestica*, *Pyrus communis* and *Prunus persica*. The volumetric flow rate was calculated by multiplication of measured mass flow velocity with the median area of ten sieve elements. All parameters were compared between phytoplasma infected and non-infect trees within each plant species.

|  | **Parameter** | **Typ of analysis** | **Error**  **distribution** | **Link-Funktion** | **Chisq /**  **F-value** | ***P*-value** |
| --- | --- | --- | --- | --- | --- | --- |
| **Apple** | velocity phloem mass flow | Glmer | Gamma | inverse | *χ²*= 8.4098 | 0.004 |
|  | vol. flow rate | Glmer | Gamma | log | *χ²*= 22.579 | <.0001 |
|  | relative density | Glm | Gamma | log | *χ²*= 0.512 | 0.613 |
|  | dynamic viscosity | Glmer | Gamma | inverse | *χ²*= 0.707 | 0.400 |
|  | density | Lmer | - | - | *F*=0.606 | 0.436 |
| **Pear** | velocity phloem mass flow | Glmer | Gamma | log | *χ²*= 8.553 | 0.003 |
|  | vol. flow rate | Glmer | Gamma | log | *χ²*= 4.688 | 0.030 |
|  | relative density | Glm | Gamma | log | *χ²*= 14.146 | <.0001 |
|  | dynamic viscosity | Lmer | - | - | *F*=9.159 | 0.009 |
|  | density | Lmer | - | - | *F*=2.462 | 0.150 |
| **Peach** | velocity phloem mass flow | Glmer | Gamma | log | *χ²*= 0.922 | 0.337 |
|  | vol. flow rate | Glmer | Gamma | log | *χ²*= 5.530 | 0.019 |
|  | relative density | Glm | Gamma | log | *χ²*= 0.818 | 0.366 |
|  | dynamic viscosity | Not determined | | | | |
|  | density | Not determined | | | | |
