# Supplementary material for: Specialized 16SrX phytoplasmas induce diverse morphological and physiological changes in their respective fruit crops
Source: PLoS Pathog. 2021 Mar 25;17(3):e1009459. doi: 10.1371/journal.ppat.1009459 (PMC8023467; doi:10.1371/journal.ppat.1009459)
Supplement: S5 Table — The callose deposition at the sieve plates was visualized by DAPI staining and fluorescence microscopy from phytoplasma infected and non-infected Malus domestica, Pyrus communis and Prunus persica and compared within each plant species. (DOCX) [file ppat.1009459.s007.docx]

**S5 Table. Specification and results of generalized least square models analyzing the maximum callose fluorescence.** The callose deposition at the sieve plates was visualized by DAPI staining and fluorescence microscopy from phytoplasma infected and non-infected *Malus domestica*, *Pyrus communis* and *Prunus persica* and compared within each plant species.

|  | **Parameter** | **Typ of analysis** | **Variance structure** | ***F*-value** | ***P*-value** |
| --- | --- | --- | --- | --- | --- |
| **Apple** | Callose  fluorescence | Gls | varIdent  ~ 1 \| treatment | 2.753 | 0.097 |
| **Pear** | Callose  fluorescence | Gls | -varIdent  ~ 1 \| treatment | 5.478 | 0.026 |
| **Peach** | Callose  fluorescence | Gls | varIdent  ~ 1 \| treatment | 36.533 | <.0001 |
