# Supplementary material for: Specialized 16SrX phytoplasmas induce diverse morphological and physiological changes in their respective fruit crops
Source: PLoS Pathog. 2021 Mar 25;17(3):e1009459. doi: 10.1371/journal.ppat.1009459 (PMC8023467; doi:10.1371/journal.ppat.1009459)
Supplement: S6 Table — Concentrations of Phytohormones salicylic acid (SA), jasmonic acid-iso leucine (JA-Ile), jasmonic acid (JA), abscisic acid (ABA), 12-Oxo-phytodienoic acid (cis-OPDA) and indole acetic acid (IAA) were determined from phytoplasma infected and non-infected Malus domestica, Pyrus communis and Prunus persica leaves. Phytohormone analysed by liquid chromatography–mass spectrometry and concentrations compared between infected a non-infected trees within each plant species. (DOCX) [file ppat.1009459.s008.docx]

**S6 Table. Specification and results of linear models used for analysis of phytohormone concentrations***.* Concentrations of Phytohormones salicylic acid (SA), jasmonic acid-iso leucine (JA-Ile), jasmonic acid (JA), abscisic acid (ABA), 12-Oxo-phytodienoic acid (cis-OPDA) and indole acetic acid (IAA) were determined from phytoplasma infected and non-infected *Malus domestica*, *Pyrus communis* and *Prunus persica* leaves. Phytohormone analyzed by liquid chromatography – mass spectrometry and concentrations compared between infected a non-infected trees within each plant species.

|  | **Phytohormone** | **Typ of analysis** | **Transformation** | **Variance structure** | ***F*-value** | ***P*-value** |
| --- | --- | --- | --- | --- | --- | --- |
| **Apple** | SA | Lm | log+1 |  | 45.305 | <.0001 |
|  | JA-Ile | Lm | log |  | 16.796 | 0.0001 |
|  | JA | Lm | log+1 |  | 3.8818 | 0.054 |
|  | ABA | Lm | log+1 |  | 14.351 | 0.0004 |
|  | cis-OPDA | Lm | sqrt |  | 4.2893 | 0.044 |
|  | IAA | Lm | - |  | 0.9362 | 0.338 |
| **Pear** | SA | Lm | box-sox |  | 0.128 | 0.726 |
|  | JA-Ile | Lm | box-cox |  | 0.005 | 0.983 |
|  | JA | Lm | sqrt |  | 0.825 | 0.379 |
|  | ABA | Gls | - | varIdent  ~ 1 \| treatment | 2.487 | 0.137 |
|  | cis-OPDA | Lm | box-cox |  | 0.033 | 0.858 |
|  | IAA | Lm | log |  | 3.575 | 0.080 |
| **Peach** | SA | Lm | log |  | 7.550 | 0.016 |
|  | JA-Ile | Lm | box-cox |  | 4.695 | 0.048 |
|  | JA | Lm | log |  | 0.917 | 0.355 |
|  | ABA | Lm | log+1 |  | 0.046 | 0.834 |
|  | cis-OPDA | Lm | sqrt |  | 0.493 | 0.494 |
|  | IAA | Lm | log+1 |  | 26.48 | 0.0001 |
