# Supplementary material for: Specialized 16SrX phytoplasmas induce diverse morphological and physiological changes in their respective fruit crops
Source: PLoS Pathog. 2021 Mar 25;17(3):e1009459. doi: 10.1371/journal.ppat.1009459 (PMC8023467; doi:10.1371/journal.ppat.1009459)
Supplement: S7 Table — The phytohormone concentrations were determined for leaf material of several plant species—Malus domestica, Pyrus communis and Prunus persica. Diverse phytohormones were detected—salicylic acid (SA), jasmonic acid-iso leucine (JA-Ile), jasmonic acid (JA), abscisic acid (ABA), 12-Oxo-phytodienoic acid (cis-OPDA) and indole acetic acid (IAA). Different letters indicate significant differences between phytoplasma infected and uninfected trees compared among the species. AP = apple proliferation, PD = pear decline, ESFY = European stone fruit yellows, npl = amount plants, nl = amount leaves. (DOCX) [file ppat.1009459.s009.docx]

**S7 Table.** **Overview of mean phytohormone concentrations (ng gFM-1).** The phytohormone concentrations were determined for leaf material of several plant species - Malus domestica, Pyrus communis and Prunus persica. Diverse phytohormones were detected - salicylic acid (SA), jasmonic acid-iso leucine (JA-Ile), jasmonic acid (JA), abscisic acid (ABA), 12-Oxo-phytodienoic acid (cis-OPDA) and indole acetic acid (IAA). Different letters indicate significant differences between phytoplasma infected and uninfected trees compared among the species. AP = apple proliferation, PD = pear decline, ESFY = European stone fruit yellows, npl = amount plants, nl = amount leaves

|  | **infection status** | **SA** | **JA** | **ABA** | **JA-Ile** | **cis-OPDA** | **IAA** | **n_pl_** | **n_l_** |
| --- | --- | --- | --- | --- | --- | --- | --- | --- | --- |
| **Apple** | healthy | 125.43  (±40.23)**^a^** | 2.86  (±4.25)**^a^** | 12.88  (±3,96)**^a^** | 0.78  (±2.031)**^a^** | 29.63  (±25.18)**^a^** | 43.59  (±12.75)**^a^** | 14 | 28 |
|  | AP | 262.71  (±127.87) | 4.04  (±3,14) | 19.98  (±9.91) | 1.38  (±1.46) | 16.43  (±12.33) | 45.50  (±13.62) | 13 | 25 |
| **Pear** | healthy | 44.83  (±14.46)**^b^** | 0.95  (±0,69)**^b^** | 77.09  (±35.75)**^b^** | 0.16  (±0.13)**^ab^** | 16.93  (±10.6)**^a^** | 20.75  (±6.00)**^b^** | 5 | 10 |
|  | PD | 44.88  (±34.60) | 1.37  (±1.11) | 102.59  (±70.77) | 0.24  (±0.22) | 10.82  (±5.88) | 14.68  (±6.74) | 5 | 10 |
| **Peach** | healthy | 167.64  (±144.06)**^a^** | 1.01  (±0.98)**^ab^** | 31.39  (±5.25)**^c^** | 0.11  (±0.13)**^b^** | 73.64  (±76.66)**^a^** | 20.32  (±4,19)**^b^** | 4 | 8 |
|  | ESFY | 487.97  (±514.97) | 2.14  (±2.57) | 33.01  (±15.83) | 0.49  (±0.51) | 86.24  (±55.26) | 12.31  (±2.23) | 4 | 8 |
