# Supplementary material for: Specialized 16SrX phytoplasmas induce diverse morphological and physiological changes in their respective fruit crops
Source: PLoS Pathog. 2021 Mar 25;17(3):e1009459. doi: 10.1371/journal.ppat.1009459 (PMC8023467; doi:10.1371/journal.ppat.1009459)
Supplement: Attachment — Submitted filename: Answers to reviewer.pdf [file ppat.1009459.s010.pdf]

Response to Reviewer's Comments

Part I – Summary

Reviewer #1:

**Comment**	
In the submitted manuscript PPATHOGENS-D-20-01286, the authors quantified various morphological and physiological changes induced by different phytoplasmas belonging to the same group 16SrX in apple, pear and peach trees. Their measurements indicated that the observed changes depend on the pathosystem. The authors' postulate is that apple tree (and not pear and peach trees) survive to phytoplasma infection thanks to morphological and physiological adaptations during the course of infection. This work is certainly of good interest for phytoplasmologists, given that symptoms and physiological changes (callose deposition, phloem sap properties, phytohormones production…) have not been detailed yet in literature. Such an extensive description will help better understand these diseases. Methodology seems appropriate, the manuscript is clearly written, and illustrations are of good quality.	
**Answer**	
Thank you.	

**Comment**	
However, the authors' conclusions are mainly based on correlations and not on demonstrations. There is no mechanistic study. In addition, the results, even when they are surprising (some more pronounced local symptoms in apple tree for example), are discussed in order to confirm the authors' hypothesis. Alternate hypotheses are not discussed. In other words, the reasoning often appears convoluted in order to satisfy the starting postulate.	
**Answer**	
Thank you for your constructive criticism. We agree that the discussion of our hypothesis was not well-balanced. Thus, we changed the evolutionary emphasis of the discussion to a much broader ecological context.	
Firstly, we changed the title from a hypothesis, which is not well underlined by a mechanistic approach, to one which describes the results of our investigations.	Line 1-2
Secondly, we changed the intermediate titles in the discussion to reduce the hard hypothesis.	Line 234, 282, 321
Thirdly, we included alternative statements into the discussion.	Line 322-343

Reviewer #2:

**Comment**
The authors performed a comprehensive study to investigate morphological and physiological responses of three fruit trees to the infection with different phytoplasmas. Therefore, they analyzed cellular and physiological parameters like morphological changes of the vascular system as well as physiological changes of the phytohormone level and phloem mass flow velocity, thereby using a broad range of different inventive methods. The study is new and will surely contribute to increasing the knowledge of how different plants deal with a phytoplasma infection. As fruit tree diseases caused by phytoplasma infections are causing immense annual crop losses, the identification and characterization of these morphological and physiological changes may help to understand how a plant becomes tolerant against phytoplasma. Therefore, the manuscript is of great interest for people working in this field.

**Answer**	
Thank you.	

Reviewer #3:

**Comment**
Manuscript PPATHOGENS-D-20-011286 entitled ‘Morphological and physiological adaptations of infected fruit trees provide tolerance against phytoplasma diseases’, by Jannicke Gallinger and coworkers provides a comparative description of the anatomical and physiological effects of three temperate fruit trees phytoplasmas of the taxonomic group 16SrX, namely “Candidatus Phytoplasma mali”, “Ca. P. pyri” and “Ca. P. prunorum” known as being respectively restricted to Malus, Pyrus and Prunus spp. The manuscript especially demonstrates that these plant species present contrasted responses to their specific phytoplasma species. Although previous study had described some of the anatomical changes presented in the manuscript, it is up to my knowledge the first comprehensive precise quantification of morphological and physiological changes of the vascular system in this different Rosaceae species in the same time that leaf phytohormones levels. The manuscript shows that “Ca. P. mali” -infected apples trees exhibit reductions of average leaf surface associated with a reduced phloem mass flow velocity whereas leaf shape is not affected (ratios of Figure 1A). In contrast, it is shown that Pyrus communis plants infected with “Ca. P. pyri” do not show such reduction of leaf size have and have an increased phloem mass flow velocity or other physicochemical characteristics of phloem sap such as volumetric flow rate, dynamic viscosity and refractive index. They show that Prunus persicae plants infected by “Ca. P. prunorum” have yellowing leaves poorly affected in their size and surface but do not show significant change in their phloem mass flow velocity while they exhibit a significantly reduced volumetric flow rate. For both phytoplasma-infected Pyrus and Prunus the authors measure an increase of callose deposition. The phytohormone levels in Pyrus appeared unchanged whilst increase of salicylic acid and jasmonic acid-isoleucine are mostly found in Malus and Prunus.<br>According to me the methodologies employed do not suffer flaws and future studies targeting germplasm resistance will benefit from the clarity of description of the parameters measured.

**Answer**	
Thank you.	

**Comment**
Maybe I missed it but I did not see any measurement of the total canopy surface and the total number of leaves. Does these parameters influence all the other ones? For instance, in Figure 1C, the healthy peach control looks bushy with much more leaves than the ‘Ca. P. prunorum” infected peach.

**Answer**	
We counted the leaves as you suggested. Indeed, infected apple trees had more leaves than uninfected ones. The leaf number both in pear and peach trees did not differ depending from a phytoplasma infection.	Line 130-132 and 451-452; Tab 1, Tab 3 and S2.

<table>
<tr><th colspan="2">Comment</th></tr>
<tr><td colspan="2">The data support the main author conclusion, i.e. that Malus show a morphological response certainly leading to a tolerance behaviour to ‘Ca. P. mali’. However, the authors bet that a response to infection necessarily constitutes an adaptation. It is a choice that must be made explicit. Indeed, a response to infection may be inappropriate. It depends on your point of view. A beneficial response at the individual level, such as the evolution towards tolerance, always has a cost at the population level: the population must bear the endemic presence of the pathogen. Conversely, a response harmful to the individual such as the rapid death of infected individuals has a benefit for the population, which then escapes the endemic presence of the pathogen. The pathosystems chosen and the results presented clearly illustrate these different situations which certainly depend on the different epidemiologies which should, in my opinion, be presented in the introduction and not at the end of the discussion. Therefore, the manuscript would benefit to also draw conclusions and discuss not only at the level of the individual tree but at the level of population. Indeed, the difference in the epidemiological systems, with psyllids vector migrating to other hosts for overwintering as it is the case for Apple and Peach trees or fulfilling their complete life cycle on pear for pear psyllids. This certainly has consequences on the adaptation strategies. As a consequence, infected pear trees react strongly by comparison to infected Malus and die faster upon infection. This could be seen as a way to limit pear to pear infection and reduce the polycyclic spread of the “Ca. P. pyri” epidemics. For all three pathosystems, hypovirulent phytoplasma strains have been described although the mechanisms of this hypovirulence is unknown as well as the key drivers favouring such evolution. Why some “Ca. P. mali” strains had evolved to hypovirulence in the context of a tolerant host? The authors produced enough data to propose sound interpretations on adaption strategy at the level of the individuals or plant populations with different kind of trade-offs.</td></tr>
<tr><th colspan="2">Answer</th></tr>
<tr><td>Thank you for this valuable comment. We agree with you that these aspects are very important to understand the pathosystems at the epidemiological level, but it is out of the scope of our study. The vector-interactions have been not investigated from us. As each participant of the tritrophic interaction (Vector/Phytoplasma/Host Plant) influences each other, we absolutely agree with your above mentioned ideas. In fact, our current study is focused on host plant-phytoplasma interactions. Thus, we favor to mention some of your ideas in the discussion and not in the introduction. We added a section about the differences in the epidemiology to the discussion and discussed the consequences / trade-offs of infections between individuals and at the population level. It is an interesting point, but as we investigated cultivated fruit species, which are propagated by grafting and not by heredity, we decided not to go in too much detail.</td><td>Line 322-343</td></tr>
</table>

**Comment**	
Finally, a response to infection can result from the intrinsic organogenetic structure of the host. In that case the response does not result from an adaptation but instead reflect the lack of adaptability of the host. I am not a specialist, but is there anything that differs in Apple trees vs Pyrus and Prunus regarding the vascular system morphogenesis. If yes it should be discussed somewhere.	
**Answer**	
It is an interesting and obvious idea to look for any specifications of the vascular system (especially, the phloem) among the single genera or even species. To our opinion, a possible existing intrinsic organogenetic structure within the single species can also be an (evolutionary) adaptation. It is hard to distinguish because everything in the biological nature base upon evolution.<br>We did not really found any specific studies that compared the phloem, for instance. We know that Nii described differences in ingrowth structures in the sieve elements (SEs) of the phloem among single Rosacea species and found rather similar structures in *Malus* and *Pyrus* in comparison to *Prunus* (Nii N (1993) Anatomical features of the sieve elements in the vascular bundles of Rosaceae fruit trees. Journal of the Japanese Society for Horticultural Science 62(1):55-61.). In theory, the ingrowth structures might influence the phloem mass flow because of the reduced size/volume of SEs and thus, reflect a higher resistance for the phloem mass flow. Maybe a phytoplasma colonization of the SEs might affect negatively the phloem mass flow (=plant physiology) in addition. Donghua and Xinzeng found also similar phloem structures in *Malus* and *Pyrus* in comparison to *Prunus* (Donghua L, Xinzeng G (1993) Comparative anatomy of the secondary phloem of ten species of Rosaceae. IAWA journal 14(3):289-298.). The length of the SEs in Prunus are shorter and each SE is connected to 2 to 3 companion cells (CCs) in comparison to Malus and Pyrus. In theory, the Prunus SEs have a higher flow resistance because of their shortness and the automatic increased number of sieve plates. But, that might be balanced with the higher amount of CCs per SEs. The CCs guarantee the physiological function of the SEs and a rise of CCs per SEs might enable the Prunus species to establish higher pressures, or a higher viability/turnover, for instance.<br>In total, you can find some indications but they are very speculative in terms to a possible organogenetic structure in the single Rosaceae species. Especially, if you try to link it with the pathogenesis in the single species. Because of the very speculative character, we disregarded that aspect in the discussion. We felt confirmed that phloem-focused studies for phytoplasma diseases are still underrepresented and support our motivation for the present study.	

Part II – Major Issues: Key Experiments Required for Acceptance

Reviewer #1:

**Comment**	
The three pathosystems used here are very different, and are in my opinion difficult to compare. In order to obtain clues allowing being more affirmative and demonstrative, it would be necessary to carry out an intra-specific study.	
**Answer**	
We have to contradict you as we compared indeed three very closely related pathosystems. All three plant species belong to the same plant family, the Rosaceae, and the same subfamily Spiraeoidea, comprising stone and pome fruits. The investigated three phytoplasma species belong all to the same group of phytoplasmas, the 16SrX or apple proliferation group. All members of this group are genetically closely related (Seemüller and Schneider 2004; Jarausch et al. 2019). Indeed, our results show major differences between the investigated pathosystems concerning host plant responses due to naturally occurring infections. This is remarkable and was not expected. The used methods and investigated parameters in this manuscript will build the basis for further investigations. We agree with you that future intra-specific studies using our methodology will be very interesting. Thus, we added this aspect and propose such investigations as next step in discussion.	Line 226-229

Reviewer #2:

**Comment**	
(No Response)	
**Answer**	

Reviewer #3:

**Comment**	
I do not see key experiments that are absolutely required. However, as the methodologies and data produced will be used in future studies for germplasm improvement and because these three pathogens have shown strong variations in virulence, the genetic identity of the phytoplasma strains used should be given. As MLSA and MLST schemes are available for phytoplasmas of this group it is not difficult to provide a genetic description of the strain used.	
**Answer**	
We added the requested information by citing the respective publications providing the genetic description of the used strains and by providing an overview table 4 with the respective information.	Lines 374

Part III – Minor Issues: Editorial and Data Presentation Modifications

Reviewer #1:

**Comment**	
I do not understand the following legend in tables and figures: 'Different letters indicate significant differences between phytoplasma infected and uninfected trees compared within each species'. What do the letters a and b refer to exactly?	
**Answer**	
Letters refer to statistical differences at a significance level of p<0.05. We added this information to legends. Exact p-values are reported in the supplementary material as stated in the methods section.	

Reviewer #2:

**Comment**	
Material and method section: Please indicate which buffer was used for the collection of phloem sap. Was EDTA included?	
**Answer**	
No. We didn't use any buffer for the collection of phloem sap. We used the centrifugation technique according to Hijaz and Killiny (2014) instead of the EDTA-method. We extended the paragraph and added more information on the treatment of the samples.	Line 532-541

**Comment**	
Did the authors estimate the ratio between transporting and non-transposing SE during the determination of the phloem mass flow velocity using CFDA? A statement on this – at least in the discussion section - would be interesting in regards to the interpretation of the data concerning the increased callose deposition in SEs of the phloem tissue of peach and pear trees.	
**Answer**	
That is a good comment and we considered that point also. In preliminary experiments we found false positive stained cells due to the slicing process of hand sections with the razor blade. CFDA was not fixed within the certain sieve elements. Unfortunately, we cannot estimate a ratio between transporting and non-transporting SEs.	

**Comment**	
A clearer statement regarding the tolerance of the apple tree to a phytoplasma infection based on the data generated in the manuscript in the conclusion section would be desirable. In the authors´ opinion, what is the reason for the tolerance?	
**Answer**	
We added our hypothesis to the discussion.	

Reviewer #3:

**Comment**	
Although the results, materials and methods and discussion sections were clearly written I found two weak points. Firstly, the introduction is very weak with wrong affirmations, non-adequate citations. Secondly, in the discussion, lines 306-325 do not discuss at all the results in their context but instead is a nice overview of the difference in the three epidemiological systems and should be moved to the introduction. I detailed these two points below.	
**Answer**	
We responded to these points below in detail.	

**Comment**	
Title: The title is too general and could be misleading. It refers to the tolerance of apple trees to "Ca. P. mali" but it is written "against phytoplasma diseases" which means in general but is not at all supported by the present data or other studies.	
**Answer**	
We agree and change the title.	Line 1-2

Abstract:

**Comment**	
Line 35: remove "some" and "mostly depending on tree age"	
**Answer**	
Done.	

**Comment**	
Line 40: A morphology cannot be reduced. Replace "morphology" by "vascular area"?	
**Answer**	
Done.	

**Comment**	
The abstract does not mention that callose deposition is increased in infected *Pyrus communis* and *Prunus persicae* whereas it was not observed in infected *Malus domestica*.	
**Answer**	
Done. The results regarding callose were added to the abstract.	Line 37-38

**Comment**	
Illustrations and tables: I did not face any difficulties to read the tables and figures.<br>On figure 1C: peach grafted by healthy peach inoculum looks bushy with a much more developed canopy. Does a higher number of leaves lead to leaves of smaller size?	
**Answer**	
Very good remark! We counted the number of leaves and added the comparison to the manuscript (Table 1, S2).	Lines 205

**Comment**	
I did not find the units in Table S2.	
**Answer**	
We adjusted the caption and added the units to the table.	

Author summary:

**Comment**	
Would it be better to restrict the sentences to stone and pome fruit tree phytoplasmas instead of phytoplasmas in general?	
**Answer**	
Done. We completely rephrased the authors summary.	Line 46-59

**Comment**	
Line 48: "numerous tons" is rather vague. Remove "numerous"?	
**Answer**	
Done.	

**Comment**	
Line 49: replace 'neither helpful" by "no alternative"? neither…nor should be removed (see below)	
**Answer**	
Done.	

**Comment**	
Line 50: Looking at the immense jump in understanding phytoplasma diseases last 15 years. It is a bit sharp and does not help at all to introduce the rationale of the study. Instead, would such sentence be adequate: "Whereas phytoplasma genome sequencing has stimulated the deciphering of molecular mechanisms being the phytoplasma host interactions, the process of physiological and morphological responses of plant host to phytoplasma infection is poorly understood".	
**Answer**	
Thanks, we included your more precise sentence.	Line 48-50

**Comment**	
Line 54: "phytohormone levels were measured" instead of "phytohormones were investigated"	
**Answer**	
Done.	

**Comment**	
Line 56: Those adaptations 'may' constitute an 'higher' ability to …?<br>Line 56-57: This sentence is unclear to me. The changes cannot "constitute" but "result" from a better sensing of phytoplasma infection?<br>Would it be better as: "Those anatomical and physiological changes may result from an enhanced ability of apple tree to sense phytoplasma infection which then results in .." ?	
**Answer**	
Line 56ff was rewritten. Thanks for suggestion.	Line 53-55

**Comment**	
Line 58: "responses"	
**Answer**	
Done.	

**Comment**	
Line 62: the meaning is unclear, please specify.	
**Answer**	
Sentence rewritten.	

Introduction:

**Comment**	
Line 71: Is not “might be” a bit strong for a hypothesis?	
**Answer**	
The sentence was deleted.	

**Comment**	
Line 73: ‘of plant resistance’	
**Answer**	
Done.	

**Comment**	
Lines 76-77: Very strange affirmation that these phytoplasma originated from Europe!<br>Neither reference 11 nor reference 12 support this affirmation! If I am correct, at least “Ca. P. prunorum” and “Ca. P. pyri” have been detected in the South Caucasian area (Balakishiyeva, G., Danet, J. L., Qurbanov, M., Mamedov, A., Kheyr-Pour, A., & Foissac, X. (2010). First report of phytoplasma infections in several temperate fruit trees and vegetable crops in Azerbaijan. Journal of Plant Pathology, 92(S4), 115., Zirak et al., in Iran). This geographical ares is an area of major secondary diversification of Malus, Prunus and Pyrus. ‘Ca; P. pyri” is also present with specific vectors in Eastern Asia. When strict associations processes are acting both partners are present in original areas of the plant host. For me this affirmation may be considered an Euro-centric point of view.	
**Answer**	
Affirmation was misleading; references referred to the fact that investigated phytoplasmas were closely related. We differentiated and deleted the wrong information.	

**Comment**	
Line 78: it is totally wrong to write that phytoplasmas in general have linear chromosomes. They are circular except for the three phytoplasmas studied.	
**Answer**	
Correct. We specified it in the text.	Line 73-75

**Comment**	
Lines 81 to 101: In my opinion, this might be moved to the discussion part as not necessary to introduce the rationale.	
**Answer**	
Thanks for the suggestion. We decided to keep the paragraph in the introduction, as we think it is useful to introduce the phloem and the function of the phloem. Especially most of our data cope with the influence to the vascular system.	

**Comment**	
Line 81: reference 16 does not refer to phytoplasma in general. Please use a general citation such as Lee et al. 2000 in Annual Review of Phytopathology or other ones.	
**Answer**	
Done. Reference 16 is changed to Lee et al., 2000.	

**Comment**	
Line 88: if 'most likely' could some references be cited?	
**Answer**	
We changed 'most likely' to 'expectable'.	Line 85

**Comment**	
Line 91: "secrete into phloem sap, effector proteins that circulate to phloem cells" is more correct?	
**Answer**	
Done. We changed the sentence to "It was previously shown that phytoplasmas produce and secrete effector proteins into phloem sap that circulate to distal tissues and induce physiological changes in infected host plants".	Line 88-89

**Comment**	
Line 95: citation 26 refers to palm (monocot) phytoplasmas. I would replace as it is out of the scope of the paper.	
**Answer**	
Done.	

**Comment**	
Line 105: psyllid transmission was originally described by (Carraro et al., 1998; Frisinghelli et al., 2000; Jarausch & Jarausch, 2009; Jensen et al., 1964).	
**Answer**	
Done.	

**Comment**	
Line 111: "Plant" volatile signals. Too many references cited here. Reference 40 again about palm trees is out of the scope. The seven others, although corresponding to very nice scientific publications, correspond to auto citations. Could this number be reduced to the most important ones?	
**Answer**	
Done. We deleted 3 references and focused on the most important ones.	

Results:

**Comment**	
Line 128-131 and Table 1: Does "diameter of midribs' correspond to maximum diameter of midrib as it is the case for Maximum leaf width? If yes indicate in the method section, in table 1 and in the text.The number of leaves and plants should be reminded in Table 1.	
**Answer**	
No. To enhance the comparability among the different plant species we defined a similar leaf site. Always the middle of the leaf was considered as mentioned in the material and method part.	Line 450-451
The number of leaves and plants measured is now indicated in the legends of Table 1.	

**Comment**	
Line 143: specify what is the meaning of “degraded”: disorganized, … A degradation can result from enzymatic or chemical processes.	
**Answer**	
We changed ‘degraded’ to ‘reduced sizes’.	Line 144

Discussion:

**Comment**	
Line 253: “planthopper”	
**Answer**	
Done.	

**Comment**	
Lines 257-260: This argument could be balanced?<br>Indeed, as callose deposition is not affecting all sieve elements, peach psyllids could feed on callose-free sieve elements.	
**Answer**	
Done. We included some new sentences.	Line 267-271

**Comment**	
Did the authors searched the literature for callose deposition in apple in response to other pathogens or when apple trees are shifting to winter dormancy? In general, are apple trees producing as callose as pear trees?	
**Answer**	
For apple and pear trees we did not found any specific data within the literature.	

**Comment**	
Lines 264-266: Although I do not contest the findings cited here, it is rather surprising.<br>Could the list of unaffected phloem metabolites? Was level of maltose, malate, acetate and glyoxylate measured?	
**Answer**	
We found that the composition of phloem centrifugates of infected P. persica was not sig. different from uninfected P. persica trees. The composition metabolites were analyzed with multivariate statistics (PERMANOVA). Therefore, it is entirely possible that levels of single metabolites differ between infected and uninfected trees. We added additional sentences with recent finding.	line 277-281

**Comment**	
Lines 306-325: This part is not a discussion at all but a review. Most of it would be more useful to introduce the pathosystems in the Introduction section.	
**Answer**	
This part of the discussion was reduced and better integrated.	Line 215-229

Material and methods:

**Comment**	
Line 346: 'specify if "kirchensaller mostbirne" is a Pyrus communis or Cydonia rootstock.	
**Answer**	
Done.	

**Comment**	
A table reminding biological and geographical origin including date of isolation and genetic identity of inoculated phytoplasmas should be given.	
**Answer**	
Done. Table 4	

**Comment**	
Line 347-354: Were these strains protected from infection (virus, psyllids, ...) or maintained in open fields ?	
**Answer**	
Sources of phytoplasma strains were cultivated in insect safe houses. We add this fact to the description.	

**Comment**	
Line 356-376: Is this method original or previously publish. Please summarize in what the Doyle and Doyle method was modified.	
**Answer**	
The applied method is a variant with several small adaptations of the CTAB extraction method found in the EPPO standard PM 7/62 (3) 'Candidatus Phytoplasma mali', 'Ca. P. pyri' and 'Ca. P. prunorum', Appendix 1, diagnostic protocol for the detection of 16SrX phytoplasmas and EPPO standard PM 7/133 (1) Generic detection of phytoplasmas, Appendix 1. Appendix 1 describes an optimization of a method described by Doyle & Doyle (1990).	line 397-401
Due to the mentioned numerous modifications for extraction of DNA from woody plants, the present description of the applied method already is a summary of these modifications from the EPPO protocol.	

**Comment**	
Line 358: "apex" instead of "top"?	
**Answer**	
Done.	line 402

**Comment**	
Line 366: "shaken" instead of "shook"?	
**Answer**	
Done.	line 411

**Comment**	
Line 359: equal volume or 0,6 volume of isopropanol?<br>It seems to me that equal volume will enhance co-precipitation of proteic compounds.	
**Answer**	
Equal volume of isopropanol is used. This ratio is a frequent practice, since recommended in the EPPO standard PM 7/62 (3) 'Candidatus Phytoplasma mali', 'Ca. P. pyri' and 'Ca. P. prunorum', Appendix 1, diagnostic protocol for the detection of 16SrX phytoplasmas and EPPO standard PM 7/133 (1) Generic detection of phytoplasmas, Appendix 1 - DNA extraction - CTAB procedure (modified from Doyle & Doyle, 1990). Protein compounds precipitate primarily by adding chloroform and subsequent centrifugation step.	

**Comment**	
Line 387: why using 46 cycles while negativity threshold is Cq 30?	
**Answer**	
Cycling parameters used are based on the EPPO standard PM 7/133 (1) Generic detection of phytoplasmas, Appendix 3 – Real-time PCR for the generic detection of phytoplasmas (Christensen et al., 2004). Negativity threshold was determined by the use of a cloned 16S rDNA standard and negative controls (healthy trees).	line 440-441

**Comment**	
Line 39: remove ")" after µl.	
**Answer**	
Done.	line 440-441

**Comment**	
Line 431: healthy is improper, the opposite of infected is uninfected, wherea healthy is the opposite of diseased.	
**Answer**	
Done.	line 478

**Comment**	
Line 485: why such plurals 'saps' Does it means that it is a total of xylem + phloem sap ?<br>If yes it must be mentoned for clarity.	
**Answer**	
Plural was a mistake, but we added a statement, that significant contamination of phloem sap samples is unlikely but can't be excluded in total.	Line 537-539
