# Supplementary material for: Specialized 16SrX phytoplasmas induce diverse morphological and physiological changes in their respective fruit crops
Source: PLoS Pathog. 2021 Mar 25;17(3):e1009459. doi: 10.1371/journal.ppat.1009459 (PMC8023467; doi:10.1371/journal.ppat.1009459)
Supplement: Attachment — Submitted filename: Answers to reviewer - resubmission_final.docx [file ppat.1009459.s011.docx]

**Response to Reviewer's Comments

Part I – Summary**

**Reviewer #1:**

| **Comment** | |
| --- | --- |
| The manuscript titled “Specialized 16SrX phytoplasmas induce diverse morphological and physiological changes in their respective fruit crops” submitted by Gallinger and colleagues describes physiological and morphological characterization of diseases caused by phytoplasmas 16SrX group in Rosaceae tree crops and suggests a link between the physiological responses to the infection and the differential survival between the crops. The authors identified that apples, pears and peaches respond differently to the disease and suggested that apples, which have better survival rate, display several unique responses such as reduced leaf size, alteration of the leaf vascular morphology, increased number of leaves per tree, accumulation of stress-associated phytohormones and reduction in the velocity of phloem mass flow. In addition, callose accumulation in the vascular bundles is reduced in apples compared to peaches and pears. The authors suggested that these unique adaptations to the infection might partially explain why apples display higher tolerance to the disease compared to the other tree crops.  Diseases caused by fastidious phloem limited pathogens have devastating effects on many tree crops, but mechanistic understanding of such diseases is lacking because of the limitations in the study of these pathogens and tree crops. While the authors attempted to explain how the altered physiological responses to the bacteria in different tree crops affects disease manifestation, the data provided in the study is mainly descriptive and mechanistic understanding of these diseases was not supported by any experimental evidence. The experimental design of the study makes it difficult to understand whether the observations made are pathogen related or host related. In addition, sampling was limited to one time point and in some cases to a small number of leaves per plant which can significantly affect the understanding of disease progression. | |
| **Answer** | |
| In order to understand diseases in a mechanistic approach you need to investigate model organisms, which you may experimentally manipulate in different ways. But this is not the approach of our study, as we here compare closely related but highly specialised 16SrX phytoplasmas and interactions with their also closely related host plants. Goal of the study is to gain information on commonalities and differences of the specific systems. A mechanistic approach is not possible without information in the natural systems on the interaction of a specific phytoplasma with the vascular system and leaf phytohormone levels. Only after knowing the interactions, we can address the question if it is pathogen- or host-related. It is important to note that the 16SrX-phytoplasma systems are difficult to investigate due to some specific characteristics of phytoplasma-host interactions. First of all, phytoplasmas are obligate cell parasites as they lack many genes that encode important metabolic functions such as amino and fatty acid synthesis and therefore, they need to consume essential metabolites from their plant hosts. It is not possible to cultivate them on axenic media. Their natural host plants are perennial plants, which are difficult to cultivate and need some years of growing prior to experiments. And finally, in most cases the disease is deadly for the host within a specific and often short time span causing additional problems with measurements and experiments. E.g. in peach trees phytoplasma infection caused very fast death of the plants. Moreover, additional impairment of the trees due to removing too many leaves was avoided. Because of a definite vegetation period in Central Europe with distinct phases of severity of symptoms, we must focus to investigate just one time point.  Generally, your critic is right and address an important aspect. However, before we are able to address mechanistic approaches, we need to understand the pathogen-host interaction in the natural system. If we know the influences of the pathogens on morphological and physiological parameters related to plant defense, we will found the basis for mechanistic approaches. The consortium of the authors plans to ground a new project on the results of this study, in which we will use model plants like tobacco or *Catharanthus* for comparison and manipulation of several traits. |  |

**Reviewer #2:**

| **Comment** | |
| --- | --- |
| Manuscript PPATHOGENS-D-20-02430 entitled ‘Specialized 16SrX phytoplasmas induce diverse morphological and physiological changes in their respective fruit crops’, is a new submission of the previously submitted manuscript PPATHOGENS-D-20-011286 by Jannicke Gallinger and coworkers. It provides a comparative description of the anatomical and physiological effects of three temperate fruit trees phytoplasmas of the taxonomic group 16SrX, namely “Candidatus Phytoplasma mali”, “Ca. P. pyri” and “Ca. P. prunorum” known as being respectively restricted to Malus, Pyrus and Prunus spp. The manuscript especially demonstrates that these plant species present contrasting responses to their specific specialized phytoplasma species. The manuscript shows that “Ca. P. mali” -infected apples trees exhibit reductions of average leaf surface associated with a reduced phloem mass flow velocity whereas leaf shape is not affected. In contrast, it is shown that Pyrus communis plants infected with “Ca. P. pyri” do not show such reduction of leaf size and have an increased phloem mass flow velocity or other physicochemical characteristics of phloem sap such as volumetric flow rate, dynamic viscosity and refractive index. They show that Prunus persicae plants infected by “Ca. P. prunorum” have yellowing leaves poorly affected in their size and surface but do not show significant change in their phloem mass flow velocity while they exhibit a significantly reduced volumetric flow rate. For both phytoplasma-infected Pyrus and Prunus the authors measure an increase of callose deposition. The phytohormone levels in Pyrus appeared unchanged whilst increase of salicylic acid and jasmonic acid-isoleucine are mostly found in Malus and Prunus. According to me, the methodologies employed do not suffer flaws and future studies targeting germplasm resistance will benefit from the clarity of description of the parameters measured. By comparison to the previous submission and in response to my comment the authors added data indicating the number of leaves (Lines 130-132 and 451-452; Tab 1, Tab 3 and S2) that show an increase in infected apple trees while it was unchanged in peach and pear trees. I previously indicated that in my opinion the previous manuscript suffered a lack of discussion about the balance between the gain resulting of an increased tolerance in malus trees at the level of individuals and its cost at the level of the propagation of the phytoplasma in tree population. The authors have convincingly addressed this point by discussing it lines 322-343 of the discussion. In response to my final comment : “Finally, a response to infection can result from the intrinsic organogenetic structure of the host. In that case the response does not result from an adaptation but instead reflect the lack of adaptability of the host. I am not a specialist, but is there anything that differs in Apple trees vs Pyrus and Prunus regarding the vascular system morphogenesis ? If yes it should be discussed somewhere.” The authors did not enrich the manuscript discussion. The authors however mentionned that Nii (1993) described differences in ingrowth structures in the sieve elements (SEs) of the phloem among single Rosacea species and found rather similar structures in Malus and Pyrus in comparison to Prunus (Nii, 1993) that was confirmed by Donghua and Xinzeng (1993) that also conclude about the length of the sieve elements in Prunus being shorter and connected to more companion cells (CCs) in comparison to Malus and Pyrus. In theory, the Prunus SEs have a higher flow resistance because of their shortness and the automatic increased number of sieve plates. But, that might be balanced with the higher number of CCs per SEs. The CCs guarantee the physiological function of the SEs and a rise of CCs per SEs might enable the Prunus species to establish higher pressures, or a higher viability/turnover, for instance.” Although I agree that the studies are scarce, it could be interesting for the reader to mention the above information somewhere. | |
| **Answer** | |
| Thank you. We added a new part about the sieve element differences of several Rosaceae genera (Pyrus, Malus, Prunus) to the discussion. | Line 233-244 |

**Reviewer #3:**

| **Comment** | |
| --- | --- |
| The revised version of the manuscript submitted by Gallinger and colleagues is now convincing. The manuscript has been seriously revised. I thank the authors to have taken my previous remarks into account and to have responded to them either with a significant correction of the text or by convincing their point of view. Moreover, all the remarks made by the other reviewers also seem to have been taken into account. The entire manuscript is now in a less speculative form and the discussion thread is more result-based. The change of title seems appropriate. The results are described in a factual manner, and replaced in an ecological context. The last paragraph of the discussion remains open, and the population-wide impact is clearly envisioned. Most important in my opinion is the change in the form of the discussion which is more moderate with mention of alternative hypotheses. In the present form, no doubt that this extensive work on characterization of changes induced by phytoplasmas on these three fruit trees will be of great interest for phytopathologists working with sap-limited bacteria. Future research directions are also interesting. | |
| **Answer** | |
| Thank you very much! |  |

**Part II – Major Issues: Key Experiments Required for Acceptance**

**Reviewer #1:**

| **Comment** | |
| --- | --- |
| 1. The study is almost solely descriptive. The only actively conducted experiment that was done in the study was inoculating the plants. While the information provided in this study is important and should be published it does not provide any evidence regarding disease mechanism and how or why the different crops respond differently to phytoplasma infections. | |
| **Answer** | |
| It is correct that the study comprises data that are mainly descriptive if you consider them individually. As our main interest was to investigate the influence of the different phytoplasma infections on the plant/vascular system of their respective host plants. the merge of various single parameters, helps to understand the interactions of different defense mechanisms and will contribute to explain the pathogenesis. Thus, the main benefit of our study is to put the single parameters in a wider context in order to gain more insight in the pathosystem, e.g. by presenting the summary table and a careful discussion.  Of course, we cannot sufficiently explain the disease mechanism or why they respond differently. The complexity of those questions need many more studies. Nevertheless, we delivered parts for the complex puzzle about the phytoplasma pathogenesis to give a possible assistance for upcoming ideas. |  |

| **Comment** | |
| --- | --- |
| 2. The distribution of phloem-limited bacteria and manifestation of disease is known to be uneven between different leaves in each tree. I therefore find the number of leaves per tree which were sampled for mass flow measurements and phytohormone analyses to be a bit low (two to four per plant).  Why the authors pooled only four leaves from each tree for phytohormone analyses? Did these leaves display similar symptoms? How the leaves were chosen for sampling (was it completely random?)? Have the authors distinguished between young and mature leaves? | |
| **Answer** | |
| We only used randomly collected mature leaves with similar symptoms. That is stated now in the M+M Part. We checked the colonization status of sampled leaves by titer determination, see Table 1. In case of apple, we used only two leaves per tree (technical replicates) but more individuals (biological replicates). The infection procedure of apple trees is more successful compared to pear and peach trees. In particular, the peach trees died very fast upon phytoplasma infection. Additional impairment of the trees due to removing of too many leaves was necessary to avoid. | Table 1; Line 127-129  M+M; Line 438 |

| **Comment** | |
| --- | --- |
| 3. The authors compared three different crops inoculated by three different 16SrX phytoplasma strains. I am assuming that these analyses were made this way because of restricted host range of each of the bacteria. With that in mind I still find the comparison between the diseases highly problematic since both the host and the pathogen are different in each system. That makes it difficult to determine whether the observed phenotypes are a result of differential response to the pathogen by the host or of differences in virulence between the different bacteria. This point was made during the previous review cycle of this manuscript and the authors claimed that phylogenetically, the bacterial strains are closely related to each other. I do not think this response properly addresses the reviewer’s comments.  Closely related species of various pathogens display high variation in virulence mechanisms, virulence intensity, host range and tissue specificity and therefore the assumption that phylogenetically related species will cause the same disease is incorrect. | |
| **Answer** | |
| We did not make the assumption that phylogenetically related species will cause the same disease. We are talking on three different diseases (PD, ESFY, AP) with differences and similarities in disease symptoms. However, the aim of our study was to compare three closely related but highly specialized 16SrX phytoplasmas causing different diseases and investigate the interactions with their also closely related natural host plants, for gaining information on commonalities and differences of the specific natural pathosystems. As it is known that single strains of the same bacterium can vary in many factors such as virulence, we selected virulent strains of each phytoplasma species for comparison (Table 4). In the presented study we found many differences regarding physiological and morphological defense strategies, which we discussed against the background of the three different systems, as it is important to understand how a specific system may react to a specific stress (here: phloem-dwelling phytoplasma). For finding mechanistic explanations, the different plant defense strategies we could identify (phytohormone titers, callose deposition, etc.) have to be proven in model systems in the future. But only if we know the processes in natural pathosystems, we can expand the research to e. g. model systems for understanding underlying mechanisms. |  |

| **Comment** | |
| --- | --- |
| 4. According to the method section the sample timing was completely different for each crop. Pear, apple, and peach trees were infected with the bacteria for five, two and one years when samples were taken, respectively. I am also assuming (it is not stated in the text) that the trees were of different ages and were not kept in similar conditions and therefore were exposed to different biotic and abiotic stressors which affect their physiology.  If the authors aspire to compare between the hosts, they should inoculate and sample the plants at the same time and ideally collect several time points to assess how their physiological parameters change during disease progression. | |
| **Answer** | |
| As indicated in the M&M section, the plants representing the three investigated genera of fruit trees (Malus, Pyrus, Prunus) had different age and inoculation times. This was necessary for the cultivation of potted trees belonging to different species and differences in the tolerance of the host plants for their specific phytoplasmas. An infection of pear trees with PD phytoplasma needs some years if it could be clearly detected, and an apple tree at least 2 years after infection with AP phytoplasma before proper testing is possible, while in peach trees the ESFY phytoplasma is disrupting severe physiological processes causing many plants to die within one year. Beside this, potted Prunus trees are difficult to cultivate longer than one year without changes in fitness, while apple and pear trees could be cultivated the same way for many years in perfect condition. Thus, we tried to use each plant species in the best condition possible and with stable infection rate. Both infected and uninfected trees of the same species have been in the same age for proper comparison. We are sorry, but your assumption is wrong as all plants have been cultivated in the same greenhouse of JKI under exactly same biotic and abiotic conditions over the whole cultivation time. Thus, it is impossible to implement your further suggestions for cultivation. I guess that you are not trained in fruit tree growing, but please believe us that we know what we are doing.  For us, the core conclusion of the discussion was that we were forced to simplify our study in a way that (1) we defined the established disease as basal condition for a reliable comparison and, thus, compared the situations in fully diseased plants/trees;. (2) We hypothesized that the phytoplasma infection is the dominant effect on the vascular plant physiology and focused on that. The knowledge about the plant physiology following a phytoplasma infection is still limited, especially in terms of vascular physiology. Hence, our study provides new data and insight in this pathosystem, even if it remains somehow descriptive. To our knowledge, there is no comparable study published and the limited-data-situation motivated us to perform the study in despite of the restrictions in the experimental design you addressed. |  |

| **Comment** | |
| --- | --- |
| 5. Bacterial titers in the inoculated trees should be stated in the main text and not in the supplementary material. | |
| **Answer** | |
| Done. The bacterial titers were now mentioned in the text. | Table 1, Line 127-129 |

**Reviewer #2:**

| **Comment** | |
| --- | --- |
| I do not see key experiments that are absolutely required. In response to the request I made about a better description of the phytoplasma isolate used in this study, the authors provided the description of the isolates used in an additional table (table 4). | |
| **Answer** | |
| Thank you. |  |

**Reviewer #3:**

| **Comment** | |
| --- | --- |
| No key experiment required. | |
| **Answer** | |
| Thank you. |  |

**Part III – Minor Issues: Editorial and Data Presentation Modifications**

**Reviewer #1:**

| **Comment** | |
| --- | --- |
| No comments. | |
| **Answer** | |
|  |  |

**Reviewer #2:**

| **Comment** | |
| --- | --- |
| I found that the new proposed title better is more precise and does not mislead the reader as the previous one. All the comments I made about imprecise sentences or errors were addressed and all requested minor modifications have been made to the manuscript.  Table 4: “Source: PD infected C. pruni individuals caught in the …” what is the meaning of PD-infected ? Is it a mistake ? Maybe replace by ‘Ca. P. prunorum”-infected C. pruni ? | |
| **Answer** | |
| Thank you. Regarding table 5: Yes, thank you, we corrected the mistake: “…'*Ca*. P. prunorum' infected *C. pruni* individuals…” | Line 386 |

**Reviewer #3:**

| **Comment** | |
| --- | --- |
| Lines 75-76 : « Phytoplasmas lacking many genes….fatty acid synthesis » seems incomplete. I guess that the authors were planning to link this sentence with the following one. | |
| **Answer** | |
| Thank you, we merged both sentences. | Lines 75-77 |

| **Comment** | |
| --- | --- |
| Lines 250-251 : I would suggest to add a reference after the statement ‘As the callose deposition in response to phytoplasma infections never results in a restriction of the bacteria’ | |
| **Answer** | |
| Done. We included Pagliari et al 2017. | Line 266 |

| **Comment** | |
| --- | --- |
| Line 254 : it is not clear what are the “specific defense mechanisms” the authors are talking about here? I would suggest to be more specific. | |
| **Answer** | |
| Done. We rephrased the sentence: “… unknown specific defense mechanisms …” | Line 269 |

| **Comment** | |
| --- | --- |
| Line 255-256 : I would suggest to remove the sentence ‘Thus, the apple-phytoplasma interaction might be older and better adapted to peach and pear’ as this idea is not clearly directly linked to the previous sentence. | |
| **Answer** | |
| Done. We deleted the sentence. | Line 270 |

| **Comment** | |
| --- | --- |
| Lines 269-271 : Although these data are interesting, I would suggest to remove both sentences at the end of this paragraph, as they correspond to unpublished results. I do not think that these data are essential here. | |
| **Answer** | |
| As the paper is still under revision, we deleted the two sentences and the citation. | Line 283 |

| **Comment** | |
| --- | --- |
| Lines 285-287 : A reference is lacking at the end of the statement ‘ Moreover, typical symptoms….imbalance of phytohormones’ | |
| **Answer** | |
| Done. We added Zimmermann et al 2015. | Line 300 |

| **Comment** | |
| --- | --- |
| Lines 328-329 : ‘might have emerged’ ? | |
| **Answer** | |
| Done. | Line 340 |
