# Supplementary material for: Specialized 16SrX phytoplasmas induce diverse morphological and physiological changes in their respective fruit crops
Source: PLoS Pathog. 2021 Mar 25;17(3):e1009459. doi: 10.1371/journal.ppat.1009459 (PMC8023467; doi:10.1371/journal.ppat.1009459)
Supplement: Attachment — Submitted filename: Letter to editor.docx [file ppat.1009459.s012.docx]

Dear Editor, Dear Nian Wang,

Thank you very much for your nice and positive response of our submitted manuscript "Specialized 16SrX phytoplasmas induce diverse morphological and physiological changes in their respective fruit crops".

Regarding your concerns, we included some aspects about *Ca.* Liberibacter and Spiroplasma We also incorporated all minor comments:

Line 75: lacking changed to lack

Line 238: We changed Donghua and Xinzeng (1993) to Liu and Gao (1993)

Line 240: we changed iwa to was (that was a typing error, thank you)

# Line 265-268: We included a statement about Liberibacter. “As the callose deposition in response to phytoplasma infections never results in a restriction of the bacteria [53], it suggests that callose deposition is a costly non-functional leftover of general defence mechanisms only and for instance also found in answer to a ’*Candidatus* Liberibacter asiaticus’ infection [54].”

Line 270: “specific” was removed

Line 294-296: We included a new sentence about Liberibacter and Spiroplasma. “A broad influence of the carbohydrate situations and changed source-sink relations in plants were already described following a ‘*Candidatus* Liberibacter’ or Spiroplasma infection [61,62]. “

Line 311-314: We included a statement about SA degradation by Liberibacter. “Not surprisingly, some bacteria species utilize SA hydroxylase for a degradation of SA to catechol to suppress an adequate plant defence reaction [67] or evolved the production of effector proteins that interfere with SA regulated defence responses by activating JA pathway [68].”

Line 389: We changed “unpublished to date” to “this study”. Thank you!

New literature:

Line 741:

54.	Pitino M, Armstrong CM, Cano LM, Duan Y. Transient expression of *Candidatus* Liberibacter asiaticus effector induces cell death in *Nicotiana benthamiana*. Front. Plant Sci, 2016;7, 982.

Line 761:

61.	Killiny N. Metabolite signature of the phloem sap of fourteen citrus varieties with different degrees of tolerance to *Candidatus* Liberibacter asiaticus. Physiol. Mol. Plant Pathol. 2017;97, 20–29, doi:10.1016/j.pmpp.2016.11.004.

62.	Machenaud J, Henri R, Dieuaide-Noubhani M, Pracros P, Renaudin J, Eveillard S. Gene expression and enzymatic activity of invertases and sucrose synthase in *Spiroplasma citri* or stolbur phytoplasma infected plants. Bull. Insectol. 2007;60(2), 1543 219.

Line 776:

67.	Li J, Pang Z, Trivedi P, Zhou X, Ying X, Jia H, Wang N. ‘*Candidatus* Liberibacter asiaticus’ encodes a functional salicylic acid (SA) hydroxylase that degrades SA to suppress plant defenses. Mol. Plant Microbe Interact. 2017;30(8), 620-630.

Thank you again for your very constructive editorial and also reviewing process.

Sincerely,

Alexandra Furch
